# The KCTD family of proteins: structure, function, disease relevance

**DOI:** 10.1186/2045-3701-3-45

**Published:** 2013-11-24

**Authors:** Zhepeng Liu, Yaqian Xiang, Guihong Sun

**Affiliations:** 1 School of Basic Medical Sciences, Wuhan University, Wuhan 430072, People’s Republic of China; 2 Jinchu University of Technology, No.33 xiangshan avenue, Jingmen 448000, People’s Republic of China

**Keywords:** KCTD, BTB domain, Adaptor

## Abstract

The family of potassium channel tetramerizationdomain (KCTD) proteins consists of 26 members with mostly unknown functions. The name of the protein family is due to the sequence similarity between the conserved N-terminal region of KCTD proteins and the tetramerization domain in some voltage-gated potassium channels. Dozens of publications suggest that KCTD proteins have roles in various biological processes and diseases. In this review, we summarize the character of Bric-a-brack,Tram-track, Broad complex(BTB) of KCTD proteins, their roles in the ubiquitination pathway, and the roles of KCTD mutants in diseases. Furthermore, we review potential downstream signaling pathways and discuss future studies that should be performed.

## Introduction

The human potassium (K^+^) channel tetramerization domain (KCTD)family of proteins consists of 26 members that share sequence similarity with the cytoplasmic domain of voltage-gated K^+^ channels(Kv channels) [1-3]. The KCTD proteins have relatively conserved N-terminal domains and variable C-termini. Comparative analyses of the conserved N-terminal sequence suggest the presence of a common Bric-a-brack,Tram-track, Broad complex (BTB) domain, which is also known as the POZ domain. The BTB domain is a versatile protein-protein interaction motif that facilitates homodimerization or heterodimerization. A variety of functions have been identified for the BTB domain-containing KCTD proteins. These functions include transcriptional repression [4,5], cytoskeleton regulation [6], tetramerization and gating of ion channels [7,8], and interaction with the cullin E3 (Cul3) ubiquitin ligase complex [9,10]. In this review, we will summarize the homology between KCTD family members and some of the key features of KCTD proteins. We will also discuss the roles of mutant KCTDs in disease.

### BTB domain and homology between KCTD family members

**Figure 1:**
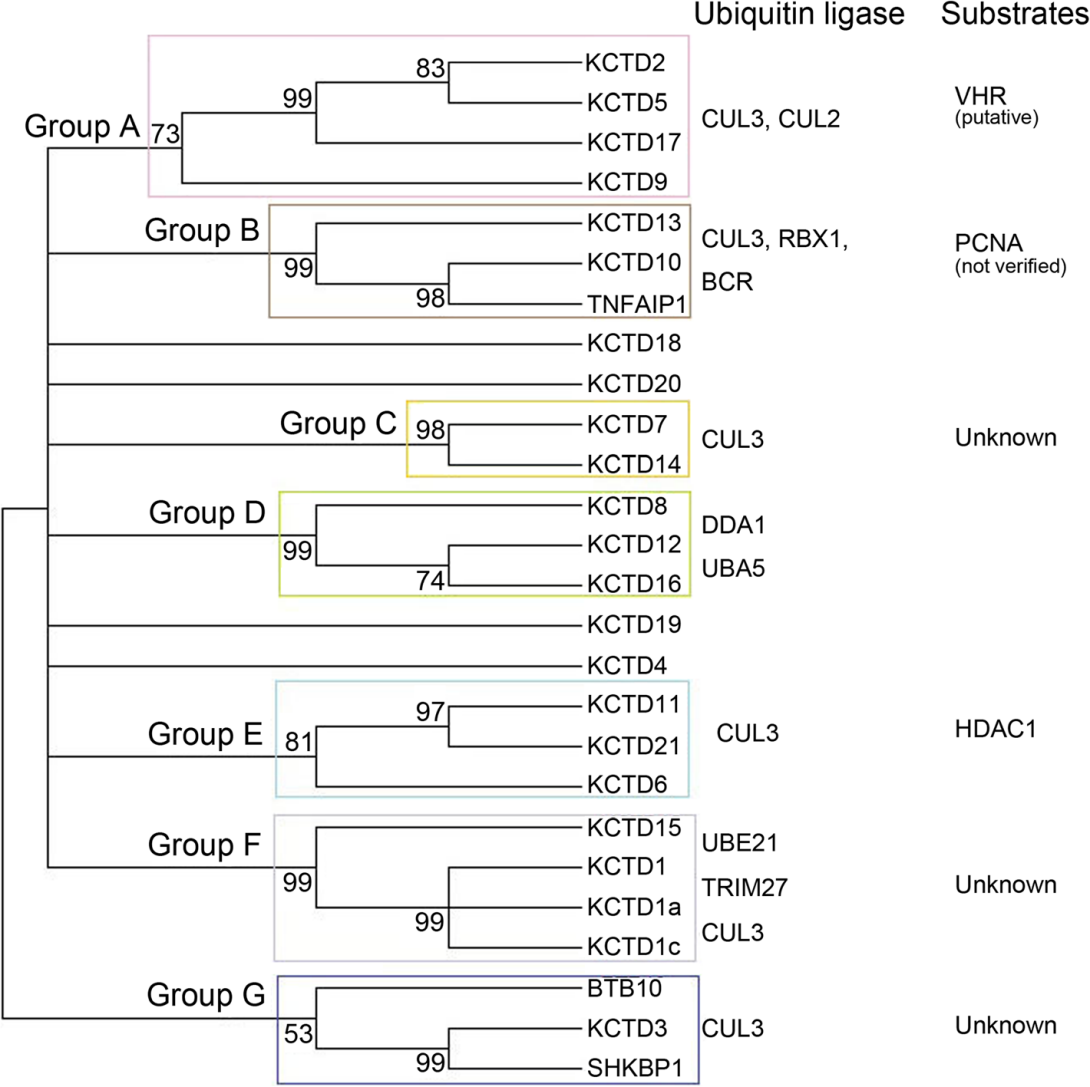
**A paralogues tree of the KCTD family proteins as cullin ligase adaptor and their substrate.** Left: A paralogues tree built using entire amino acid sequences of the KCTD family proteins; Right: the family of KCTD proteins corresponding to cullin and their substrate.

The human genome includes approximately 400 BTB domain-containing proteins. The BTB domain is a highly conserved motif of about 100 amino acids and can be found at the N-terminusof C_2_H_2_-type zinc-finger transcription factors and in some actin-binding proteins [11]. BTB domain-containing proteins include transcription factors, oncogenic proteins, ion channel proteins, and KCTD proteins [2,12-14]. Many BTB domain-containing proteins contain one or two additional domains, such as kelch repeats, zinc-finger domains, FYVE (Fab1, YOTB, Vac1, and EEA1) fingers which is a novel zinc finger-like domain found in several proteins involved in membrane trafficking, or ankyrin repeats [15]. These special domains provide unique characteristics and functions to the BTB proteins. The BTB domain facilitates protein-protein interactions between KCTD proteins to allow self-assembly or with non-BTB-domain-containing proteins to promote oligomerization [15]. The X-ray crystal structure of KCTD5 also revealed assemblies of five subunits while tetramers were anticipated [16]. A variety of functional roles of KCTD proteins have been identified by different signal pathways, including sonic hedgehog (Shh) [17-19], Wnt/beta-catenin [20], FGF [1], and GABA signaling [21-24]. Alignment of the amino acids in the potassium tetramerization domains of all known KCTD proteins demonstrates that most KCTD proteins can be divided into seven groups by amino acid sequences. The A-group contains KCTD9, KCTD17, KCTD 5, and KCTD 2. The B-group contains KCTD10, KCTD13, and TNFAIP1. The C-group contains KCTD7 and KCTD14. The d-group contains KCTD8, KCTD12, and KCTD16. The E-group contains KCTD11, KCTD21, and KCTD6. Members of the F-group include KCTD1 and KCTD15. And the final group is the G-group, which contains KCTD3 and SHKBP1 and BTB10. KCTD20, KCTD18, KCTD19, and KCTD4 do not belong to these seven groups (Figure 1). The evolutionary tree of the KCTD family proteins is similar to the group that Skoblov M et al. built [25]. We also suggest that homologous KCTD members may share similar functional roles in proliferation, transcription, protein degradation, regulation of G-protein coupled receptors and other molecular or biological processes.

### KCTD proteins as adaptor molecules

BTB-domain-containing KCTD proteins may act as adaptors for interactions between the Cul3 ubiquitin ligase and its substrates. Thus, BTB KCTD proteins may facilitate successful ubiquitination of substrate proteins [26]. Cul3 is one of seven human cullin proteins (Cul1,Cul2,Cul3,Cul4A,Cul4B,Cul5, and Cul7). Most cullins form complexes with substrate proteins by interacting with the BTB domains of adaptor proteins [3]. Thus, the BTB domain is important for the process of ubiquitination and protein degradation. Ubiquitination involves a three-step enzymatic cascade, which is initially activated by ubiquitin-activating enzyme(E1). The substrate is then transferred to ubiquitin-conjugating enzyme(E2) and is finally linked with ubiquitin ligase(E3) [27]. Various cellular functions, including cell proliferation, differentiation, apoptosis, and protein transport, involve protein ubiquitination and de-ubiquitination [28]. Bioinformatics and mutagenesis analyses have demonstrated that the best-characterized member of the KCTD family, KCTD11/REN, is expressed as two alternative variants, sKCTD11 and lKCTD11. Despite the fact that both variants possess a BTB domain in the N-terminus, only the lKCTD11 form has a complete BTB domain. Intriguingly, this has not disturbed the cul3-binding activity of sKCTD11. KCTD11/REN also mediates histone deacetylase (HDAC1) ubiquitination and degradation via cullin binding, resulting in reduced Hh/Gli signaling [18]. The *KCTD2*1 and *KCTD6* have also been found to have the same features as *KCTD11*[29]. Thus, KCTD21 and KCTD6 may also facilitate protein degradation and reduced cellular signaling due to associations with ubiquitin ligases. KCTD5 and KCTD7 have also been shown to function as substrate-specific adaptors for cullin3-based E3 ligases [3,30,31]. In addition, KCTD7 has been shown to increase potassium conductance due to increased proteasome degradation of an unidentified substrate [30]. Thus, several members of the KCTD family function as critical adaptor molecules for ubiquitin-mediated protein degradation. This function ultimately results in the modulation of important downstream signaling pathways and biological processes. As can be seen from Figure 1, cullin is fairly widely interaction with the family of KCTD proteins. In the future, this novel substrate of KCTD will help to understand the function of the complex of CUL3 –BTB.

### KCTDs and disease

KCTD proteins have essential roles in proliferation, differentiation, apoptosis, and metabolism. Improper regulation of KCTD genes has been associated with various diseases, including medulloblastoma [32], breast carcinoma [33], obesity [34,35], and pulmonary inflammation [36]. Many studies show associations between mutations in individual KCTD genes or allelic loss of KCTDs with specific diseases. For example, a homozygous mutation (R99X) in exon 2 of *KCTD7* has been described in progressive myoclonic epilepsy (PME) [37]. A second homozygous missense mutation (R94W) in exon 2 of *KCTD7 *has also been found in PME [38]. In addition, a heterozygous missense mutation (R84W) and a large heterozygous deletion of exons 3 and 4 of *KCTD7* have also been reported in patients with PME [30,31]. Allelic deletion of human *KCTD11*at chromosomal location 17p13.2 has been found in medulloblastoma [19,39]. In addition, gene copy number variants (CNVs) of *KCTD13* mapping to chromosomal location 16p11.2 are considered to be major genetic causes of macrocephaly and microcephaly. Overexpression of *KCTD13* induces microcephaly, whereas suppression of the same locus results in a macrocephalic phenotype [40]. Missense mutations in *KCTD1*occur in Scalp-ear-nipple (SEN) syndrome [41]. Single nucleotide polymorphisms(SNPs) of *KCTD10* (i5642G- > C and V206VT- > C) are associated with altered concentrations of HDL cholesterol, particularly in subjects with high levels of carbohydrate intake [42]. KCTD mutants affect proliferation, differentiation, apoptosis, and metabolism in different tissues. For example, the CNVs of *KCTD13* affect the balance of proliferation and apoptosis in neuronal progenitor cells. In addition, deletions in *KCTD11 *abrogateinhibition of Shh signaling at the outer to inner external granule layer-granule cell progenitor (EGL GCP) transitions by affecting expression of Gli1 and Gli2 [19]. Deletions in *KCASH*, *KCTD21*, or *KCTD6* block interactions with ubiquitination enzymes, preventing degradation of HDAC1. This leads to increased acetylation of Gli1 and increased Hh/Gli signaling, which drives uncontrolled proliferation and development and progression of medulloblastoma [17,39]. Not only mutant KCTD could cause diseases, but also the change of KCTD expression involved in different diseases [22,43-46]. All of the diseases related with KCTD proteins have been list in a Table 1 to make the family more convenient for further study.

**Table 1:** KCTD proteins and related diseases

	**Disease**	**KCTD - related**	**Function of KCTD proteins in disease**	**Reference**
Cancer	Gastrointestinal stromal tumor	KCTD12	biomarker	Ref. [43]
		KCTD10	prognostic biomarker	
	Medulloblastoma	KCTD11	Suppress Histone Deacetylase and Hedgehog activity in medulloblastoma	Ref. [17]; Ref. [19]; Ref. [39];
		KCTD21		
		KCTD6		
Neurological disease	Progressive Myoclonic Epilepsy (PME)	KCTD7	KCTD7 mutations might be a recurrent cause of PME	Ref. [30]; Ref. [31]; Ref. [37]; Ref. [38]
	Abnormal Head Size	KCTD13	overexpression microcephaly phenotype	Ref. [40]
			underexpression macrocephaly phenotype	
Metabolic disorder	HDL cholesterol concentration	KCTD10	KCTD10 (V206VT - > C and i5642G - > C) may contribute to the variation in HDL-cholesterol concentrations, particularly in subjects with high carbohydrate intakes.	Ref. [42]
Others	Influence EPO production	KCTD2	Production of erythropoietin (EPO) was significantly inhibited when CEBPG, KCTD2, and TMEM183A were knocked down	Ref. [44]
	Live injury of HBV-ACLF	KCTD9	The overexpressed KCTD9 activates NK cell in peripheral blood and liver in HBV-ACLF, which contributes to liver injury	Ref. [45]; Ref. [46]
	Chronic Tinnitus	KCTD12	Risk modifier	Ref. [22]
	Scalp-ear-nipple(SEN) syndrome	KCTD1	missense mutation in KCTD1 causes SEN syndrome	Ref. [41]

## Conclusion

There are some features of KCTDs that have not been reviewed in this article. For example, KCTD8, -12, -12b, and-16 form functional oligomers with the GABAB receptor, resulting in the modulation of important signaling pathways [21-24,47]. In addition, the PDIP1 family members (KCTD10, KCTD13, and TNFAIP1) are tumor necrosis factor-a-inducible proteins that can stimulate the activity of DNA polymerase in DNA replication and repair pathways [48]. Furthermore, interactions between KCTD1, KCTD15, and AP-2 represses the transcriptional activity of AP-2a [13], Finally, KCTD1 has been shown to interact with PrP^C ^[49]. In the review, we summarize the BTB characteristics of the KCTD proteins, their roles in the ubiquitination pathway, and the relevance of KCTD mutations in various diseases. The review highlight the extraordinary possibility of the interaction of cullin-KCTDs to target substrates for ubiquitin-dependent degradation. If BTB-containing KCTD proteins can assemble into Cul3-based complexes, we estimate KCTD proteins can recruit substrates into ubiquitin system. We specifically discuss the role of KCTD1 in the ubiquitination pathway via interaction with cul3. We also hypothesize that KCTD1 mediate prion protein into ubiquitination signal pathway, and deregulation of the KCTD1 mediated prion protein ubiquitination might be both a cause and result of prion disease. Furthermore, we speculate that members of the same sub-groups may have similar roles in biological processes or molecular signaling pathways. We believe that further investigations into the functions of individual KCTD family members are warranted, particularly within the context of specific diseases as described here.

## Abbreviations

KCTD: Potassium channel tetramerization domain; BTB: Bric-a-brack,Tram-track, Broad complex; Cul3: Cullin E3 ubiquitin ligase; Shh: Sonic hedgehog; E1: Ubiquitin-activating enzyme; E2: Ubiquitin-conjugating enzyme; E3: Ubiquitin ligase; HDAC: Histone deacetylase; PME: Progressive myoclonic epilepsy; CNV: Copy number variant; SEN: Scalp-ear-nipplesyndrome; SNP: Single nucleotide polymorphism; EGL GCP: External granule layer-granule cell progenitor; KCASH: KCTD containing, Cullin3 adaptor, suppressor of Hedgehog; TNFAIP1: Tumor necrosis factor, alpha-induced protein 1.

## Competing interests

The authors declare that they have no competing interests.

## Authors’ contributions

ZP, YX, and GS co-wrote this review. All authors read and approved the final manuscript.
